# How Many Patients Become Functionally Dependent after a Stroke? A 3-Year Population-Based Study in Joinville, Brazil

**DOI:** 10.1371/journal.pone.0170204

**Published:** 2017-01-20

**Authors:** Lívia Mizuki de Campos, Bruna Mariah Martins, Norberto Luiz Cabral, Selma Cristina Franco, Octávio Marques Pontes-Neto, Suleimy Cristina Mazin, Felipe Ibiapina dos Reis

**Affiliations:** 1 Department of Medicine, University of Joinville Region, Joinville, Santa Catarina, Brazil; 2 Joinville Stroke Registry, University of Joinville Region, Joinville, Santa Catarina, Brazil; 3 Department of Medicine, University of São Paulo, Ribeirão Preto, São Paulo, Brazil; Medizinische Universitat Innsbruck, AUSTRIA

## Abstract

The decrease in stroke mortality will increase the burden of survivors with functional dependence (FD). The aim of this study was to evaluate how many patients become functionally dependent over 3 years after an incident event in Joinville, Brazil. The proportion of FD (defined as a modified Rankin score 3 to 5) among stroke survivors from the Joinville Stroke Registry was assessed using a validated telephone interview. Incidence of FD after stroke in Joinville in one year was 23.24 per 100,000 population. The overall proportion of FD among stroke survivors at discharge was 32.7%. Of 303 patients with first-ever ischaemic stroke (IS), one-third were FD at discharge, and 12%, 9% and 8%, respectively at 1, 2 and 3 years. Among 37 patients with haemorrhagic stroke (HS), 38% were dependent at discharge, 16% after 1 and 2 years and 14% after 3. Among 27 patients with subarachnoid haemorrhage (SAH), 19% were dependent at discharge and 4% from 1 to 3 years. Among IS subtypes, cardioembolic ones had the worst risk of FD. (RR 19.8; 95% CI: 2.2 to 175.9). Our results showed that one-third of stroke survivors have FD during the first year after stroke in Brazil. Therefore, a city with half a million people might expect 120 new stroke patients with FD each year.

## Introduction

Stroke is a major public health problem and a leading cause of years of life lost worldwide [1]. From 1990 to 2013, the Global Burden of Disease Study Group showed a 23% stroke mortality rate decrease across the world [1]. This reduction is mainly due to the decline in early ischaemic stroke (IS) case-fatality rates, which are responsible for over two-thirds of all types of stroke [2]. An increase in the number of stroke care units, effectiveness of intravenous thrombolysis and, more recently, of mechanic thrombolysis are the main motives for the reduction in rates [3], even though it has been asymmetric between high- and low/medium-income countries [1,2]. As a consequence of this case-fatality decrease, a growing prevalence of disabled stroke survivors exposes the suffering of patients, their families, and the burden of stroke in the population at large [1].

Worldwide, the burden of ischaemic and haemorrhagic stroke (HS) increased significantly between 1990 and 2010, in terms of the absolute number of people with IS and HS (37% and 47% increase, respectively), number of deaths (21% and 20% increase), and disability adjusted-life years (DALYs) lost (18% and 14% increase). Over the past two decades in low-income and middle-income countries, a significant increase of 22% in the incidence of HS was noted and a 6% non-significant increase in the incidence of ischaemic stroke. Mortality rates for ischaemic stroke fell by 14% and DALYs lost by 17% [1,4].

Among stroke survivors, functional impairment has a huge impact on patients’ quality of life, leading to financial and emotional burden for their families and to a substantial net financial burden for the health-care system [2,5–7]. Approximately 35% of survivors with initial paralysis of the leg do not regain useful function and 20 to 25% of all survivors are unable to walk without full physical assistance [8]. In the US, stroke is the most frequent cause of adult-onset disability and the cost of related care is among the fastest growing expenses for Medicare [9].

In Brazil, results from a community-based survey conducted in 2013 showed that 25% (568,000 / 2,231,000) of stroke cases remain with severe disabilities [10]. However, data from population-based studies are very scarce in low- and middle-income countries. To address this gap of knowledge, we measured the proportion of functional dependence after stroke over a 3-year period in Joinville, Brazil, and identified risk factors for functional dependence among ischaemic stroke patients.

## Materials and Methods

Joinville is a city in Southern Brazil with an area of 1,130 km^2^ and 516,288 inhabitants, according to the last national census. The city has four hospitals (one with a stroke unit) and one state-run institutional care facility [11]. The Joinville Stroke Registry is an ongoing population-based registry databank started in 2005 and supported by law since 2013. The registry uses the methodology proposed by Sudlow and Warlow [12] as well as the Stroke-Steps modular programme proposed by the WHO (first step for all hospital cases, second step for checking of death certificates and third step to ascertain mild events) [13]. The detailed methods of cohort recruitment and data collection procedures have been described elsewhere [14].

The study population included all patients who had a first-ever stroke between October 1, 2009 and September 30, 2010. These patients were followed until September 30, 2013. The stroke diagnosis followed previously defined criteria [15]. Every day, three research nurses, on a face-to-face basis, interviewed the patients and relatives at emergency departments of all city hospitals. They registered the demographic, cardiovascular risk factors and diagnostic work-up results. The attending neurologist informed the study nurse about IS clinical stroke syndrome Oxfordshire Community Stroke Project criteria) [16] and pathophysiological diagnosis (Trial of Org 10172 in Acute Stroke Treatment; (TOAST criteria) [15]. The stroke investigation routine followed the guidelines of the Brazilian Society of Cerebrovascular Diseases [17]. The final stroke subtypes classified by the TOAST and Bamford criteria were made by respective neurologist in all hospitals.

Education was stratified in years of schooling. The economic strata followed a classification used by the ABEP (Brazilian Association of Research Enterprises), which takes into account household characteristics, such as possession and quantity of durable goods, number of bathrooms, employment of domestic workers and educational level of the head of household. Each item receives a score and the sum of scores is then associated to an economic stratum—A, B1, B2, C1, C2, D and E. It estimates the purchasing power of individuals and urban families. According to Brazilian Criteria of Economic Classification based on year 2013 National Household Sample Survey, income estimations for social-economic strata per year in US dollars for each of following classes were: Class A = 64,020; B1 = 27,468; B2 = 19,980; C1 = 8,256; C2 = 4,572; D–E = 2,016. The Brazilian gross domestic product per capita at purchasing power parity according to World Bank was US 14,997 per year in 2013 [18].

Stroke severity was defined at hospital admission according the National Institute of Health Stroke Scale (NIHSS) [15] and classified as mild (score of 1 to 4), moderate (score of 5 to 14), moderate/severe (score of 15–20) and severe (score of >21). Comorbid conditions were obtained from medical records and comprehended hypertension, diabetes, dyslipidemia and cardiopathy. Self-reported physical activity data were collected using the short version of the International Physical Activity Questionnaire (IPAQ-S), which asks participants to report activities performed for at least 10 minutes during the last 7 days.To measure functional dependence, we adopted the modified Rankin scale (mRS) which ranges from 0 (no symptoms) to 6 (death) [19]. Patients with 0 to 2 points were classified as independent and with 3 to 5 as dependent. A previously trained nurse called all patients or relatives at 12, 24 and 36 months after the stroke event, using a validated Brazilian version of the Rankin scale interview for telephone assessment [20].

We performed descriptive statistics to show baseline characteristics and proportion of dependence (Rankin score 3 to 5) for IS, HAS and subarachnoid haemorrhage (SAH) over 3 years after hospital discharge.

Taking into account that different types of stroke have distinct pathophysiological and aetiological mechanisms, which may affect prognosis and outcomes, we decided to analyse dependence only among IS patients. The reason for this choice is that ischaemic stroke is by far the most frequent event. Therefore, we performed statistical analysis for risk factors only for this group. Crude and adjusted relative risks (RR) for dependence at 12, 24 and 36 months were statistically assessed by Poisson regression model using Statistical Analysis System software version 9.2 with PROC GENMOD (SAS Institute Inc, Cary, NC) [21,22]. The Poisson model was adjusted for the variables age, gender, years of education, social class, race, hypertension, diabetes, smoking, dyslipidemia, cardiopathy, anticoagulant and anti thrombotic use, physical activity, NIHSS, TOAST and Bamford. The statistical significance of the results was tested using a 95% confidence interval, and a p-value <0.05 (two-sided) was considered significant.

The study was approved by the Research Ethics Committee of the University of the Region of Joinville, Santa Catarina, Brazil, under Protocol N. 616.055.

## Results

We identified 727 patients with stroke from October 1, 2009 to September 30, 2010. Of those, 320 were excluded based on the following criteria: 225 had prior strokes, 63 were Transient Ischaemic Attack (TIA) events, 9 patients died in the first 24 hours and 23 patients moved to other cities. Thus, the initial cohort had 407 patients. We lost 40 individuals over 3 years, 30 due to recurrent stroke and 10 to loss of follow-up. Among the remaining 367 patients, 83% (303/367) had IS, 10% (37/367) HS and 7% (27/367) SAH.

The mean age was 63 ± 16 years old, 56% were males and 91% Caucasians. Most of the patients had less than 8 years of education (68%) and 72% belonged to lower social classes (C to E). Hypertension was the most frequent premorbid risk factor (70%). Almost one-third of patients practiced physical activities, 4% had previously used anticoagulant therapy, 27% anti-thrombotic drugs and 22% were current smokers. As expected, the median National Institute of Health (NIH) scale was higher among HS and SAH patients. Among IS patients, 38% presented with a PACS syndrome and 28% had the final diagnosis of small-artery occlusion. Table 1 shows these baseline characteristics.

**Table 1:** Baseline characteristics of patients, risk factors, clinical severity and diagnosis.

		Total (N = 367)	IS (n = 303)	HS (n = 37)	SAH (n = 27)
Baseline characteristics					
Age, mean (SD)		63.4(16.2)	64.9(15.5)	58.5(19.8)	52.8(14.0)
Male		207(56.4)	174 (57.4)	21 (56.7)	12 (44.4)
Caucasian		333 (90.7)	274 (90.4)	35 (94.6)	24 (88.9)
Education (years)		*	**		***
	< 8	245 (67.7)	211 (70.3)	23 (62.2)	11 (44.0)
	≥ 8	117 (32.3)	89 (29.7)	12 (32.4)	14 (56.0)
Social Class^β^					
	A	9(2.4)	8 (2.7)	0 (0.0)	1 (3.7)
	B1	8 (2.2)	7 (2.3)	1 (2.7)	0 (0.0)
	B2	85 (23.2)	70 (23.1)	10 (27.0)	5 (18.5)
	C1	97 (26.4)	82 (27.1)	6 (16.2)	9 (33.4)
	C2	93 (25.3)	78 (25.7)	9 (24.3)	6 (22.2)
	D	73 (19.9)	57 (18.8)	11(29.8)	5 (18.5)
	E	2 (0.6)	1 (0.3)	0 (0.0)	1 (3.7)
Premorbid risk factor					
	Hypertension****	250 (69.7)	207 (69.9)	28 (77.8)	15 (55.5)
	Diabetes*****	80 (21.9)	72 (23.8)	5 (13.5)	3 (11.1)
	Dyslipidemia******	212 (61.1)	173 (60.1)	21 (61.8)	18 (72.0)
	Cardiopathy	128(34.9)	113 (37.3)	10 (27.0)	5 (18.5)
Physical activity		110 (30.0)	95(31.3)	6 (16.2)	9 (33.3)
Previous anticoagulant use		16 (4.4)	13 (4.3)	3 (8.1)	0
Previous anti thrombotic use*******		99 (27.1)	91 (30.0)	5 (13.5)	3 (11.1)
Smoking					
	Ex	109 (29.7)	94 (31.0)	10 (2.7)	5 (18.6)
	No	176 (48.0)	147 (48.5)	19 (51.3)	10 (37.0)
	Current	82 (22.3)	62 (20.5)	8 (21.6)	12 (44.4)
NIHSS,median (IQR)		6 (15.5)	5 (14)	18 (19)	16 (24.5)
NIHSS					
	Minor stroke	138 (37.6)	128 (42.2)	4 (10.8)	6 (22.2)
	Moderate stroke	91 (24.8)	78 (25.8)	10 (27.0)	3 (11.1)
	Moderate/Severe stroke	36 (9.8)	27 (8.9)	5 (13.5)	4 (14.8)
	Severe stroke	80 (21.8)	52 (17.2)	17 (46.0)	11 (40.8)
TOAST					
	SAO		86(28.4)		
	CE		79(26.1)		
	Undetermined		73(24.1)		
	LAA		58(19.1)		
	Other determined		7(2.3)		
Bamford					
	PACS		116(38.3)		
	LACS		89(29.4)		
	TACS		67(22.1)		
	POCS		31(10.2)		

Data are number of patients (%) unless otherwise indicated; IS: ischaemic stroke; HS:haemorrhagic stroke; SAH: subarachnoid haemorrhage; SAO: small-artery occlusion; CE: cardioembolic; LAA: large-artery oclusion; PACS:partial anterior circulation syndrome; LACS: lacunar syndrome; TACS: total anterior circulation syndrome; POCS: posterior circulation syndrome; unavailable data:

* 5 (1.4%)

**3(0.8%)

*** 2(0.5%)

**** 8 (2.2%)

***** 1 (0.3%)

****** 20 (5.3%)

******* 2 (0.5%)

β Social class according to Brazilian Criteria of Economic Classification based on year 2013 National Household Sample Survey.

**Fig 1:**
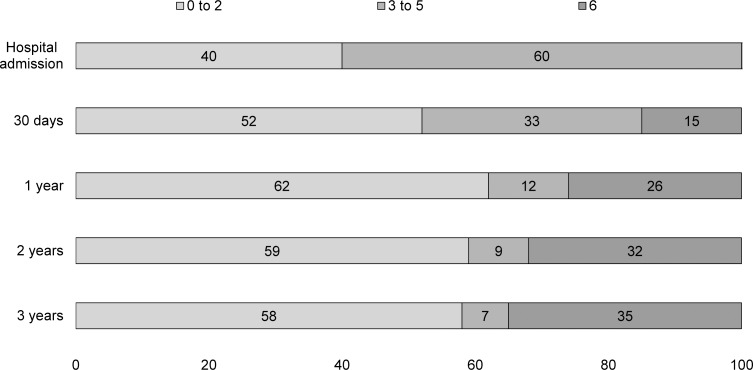
Modified Rankin Scale (mRS) scores of 303 first-ever ischaemic stroke patients. Proportions of patients after hospital admission, 30 days and 1 to 3 years in Joinville, 2008 to 2010; Rankin score 0 to 2: functional Independence, 3 to 5:functional dependence and 6:death.

**Fig 2:**
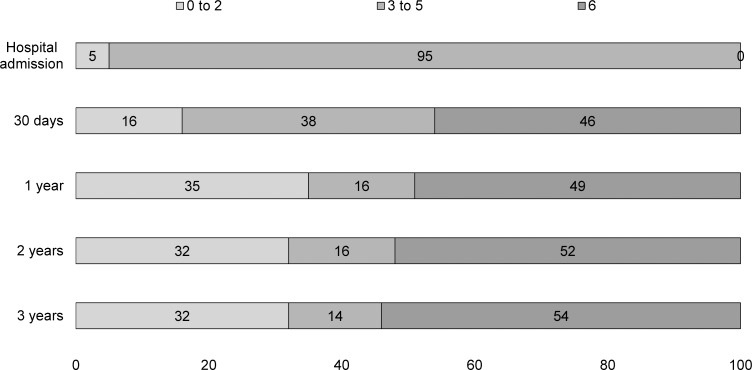
Modified Rankin Scale (mRS) scores of 37 first-ever haemorrhagic stroke patients. Proportions of patients after hospital admission, 30 days and 1 to 3 years in Joinville, 2008 to 2010; Rankin score 0 to 2: functional Independence, 3 to 5:functional dependence and 6:death.

**Fig 3:**
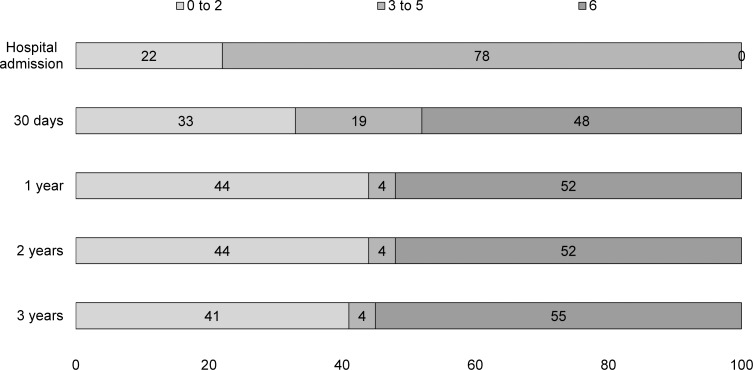
Modified Rankin Scale (mRS) scores of 27 first-ever sub-arachnoid haemorrages stroke patients. Proportions of patients after hospital admission, 30 days and 1 to 3 years in Joinville, 2008 to 2010; Rankin score 0 to 2: functional Independence, 3 to 5:functional dependence and 6:death.

Incidence of FD after stroke in Joinville was 23.24 per 100,000 population. The overall proportion of functional dependence (Rankin 3 to 5) at hospital admission was 65% (95% CI, 60.2%–70.0%), reducing to 33% (95% CI, 27.9%–37.5%) at 30 days, to 12% (95% CI, 8.4%–15.0%) in the first year, to 9% (95%CI, 6.3%–12.2%) in the second year and to 8% (95%CI, 4.9%-–10.3%) in the third year. As expected, the highest dependence proportion was found among HS patients. In all major stroke types, the functional dependence decreased more over the first year, remaining stable thereafter. Almost half of all HS and SAH and a third of IS patients died in the first year (Figs 1–3).

**Fig 4:**
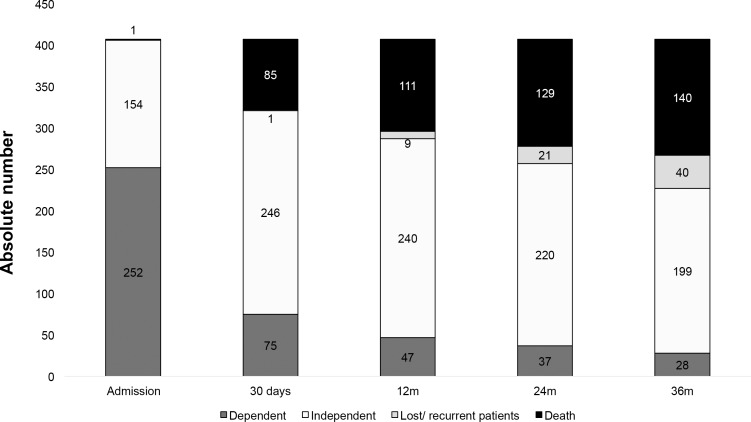
Evolution of functional status of 407 patients with all stroke types measured by Rankin scale over 3-years.

The evolution of functional status of the entire cohort (407 patients) including all stroke types measured by Rankin scale over 3-years showed a decrease in the absolute number of both dependent and independent patients while it was observed an increase in the number of patients who died (Fig 4).

The decrease of FD over the time, when we included all stroke patients, was 25% in 1 month, 17% in 12 months; 14% in 24 months and 12% in 36 months. (p = 0.0006). For IS, however, we noticed a trend for decrease, as p = 0.45 in 12 months, 0.15 in 24 months and 0.14 in 36 months. Among HS patients, there was a significant decrease all the time, from 0.084 at 12 months; 0.0163 at 24 months and 0.026 at 24 months. Finally, among SAH there was no decrease in FD proportion, since most of them died or became independent (p = 0.35; 0.48 and 0.66 at 12, 24 and 36 months, respectively).

**Fig 5:**
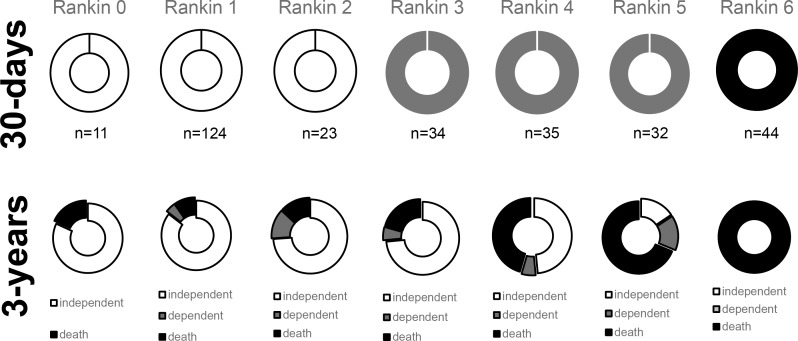
Functional status of 303 patients after incident stroke measured by Rankin scale at 30 days and 3 years.

Fig 5 shows the functional evolution of 303 patients with IS comparing Rankin score (0 to 6) from 30 days to 3 years. The 11 patients who were completely independent in 30 days, 18% (2/11) died and the rest remained independent. From 124 with one point in mRS, 85% (106/124) remained independent, 2% (6/124) were dependent and 5% (12/124) died. From 23 with 2 points in mRS, 74% (17/23) remained independent, 13% (3/23) became dependent and 13% (3/23) died. From 34 with 3 points in mRS, 74% (25/34) became independent, 11% (2/34) remained dependent and 20% (7/34) died. From 35 with 4 points in mRS, 46% (16/35) became independent, 11% (4/35) remained dependent and 43% (15/35) died. From 32 with 5 points in mRS, 16% (5/32) became independent, 16% (5/32) remained dependent and 69% (22/32) died. Comparing the evolution of independent *versus* dependent groups, we found that 85% (134) of 158 IS patients who were independent in first month remained independent whereas 46% (46) of 101 IS patients who were dependent in first month became functionally independent. The proportions of improvement were worse in HS and SAH patients. Of 13 patients with HS who were independent in first month, 85% (11) remained independent in 3 years, whereas among 12 HS patients who were dependent, only 8% (1) became independent. Of 12 patients with SAH who were independent in first month, 92% (11) remained independent in 3 years. The only one patient with SAH who were dependent in first month remained dependent in 3 years.

We analysed the variables that could be associated with functional dependence at 12, 24 and 36 months, among first-ever IS patients. In 303 of them, we found that socio-demographic characteristics, risk factors and clinical severity were not associated with functional dependence in 1 to 3 years after hospital discharge. Among IS subtypes, patients with cardio-embolic strokes had a much higher risk of dependence at 12 and 24 months. In the first year, compared to small-artery occlusion, cardio-embolic patients had an adjusted relative risk of dependence twenty-fold higher than lacunar stroke (RR 19.8, 95% CI, 2.2 to 174.8; p = 0.007). This risk decreased to 12 (1.7 to 79; p = 0.01) in the second year and to 5.3 (0.82 to 34; p = 0.08) in the third year. Other determined and large-artery occlusion IS also had an increased relative risk compared to small-artery occlusion (RR: 16.7, 95% CI 1.3 to 212.3, p = 0.03; RR 11.6, 95% CI 1.4 to 104.4, respectively. After 2 years, the risk of dependence in cardio-embolic stroke was 12 times higher than lacunar stroke (RR11.8, 95% CI 1.77 to 79). After 3 years, no variables were associated with functional dependence (Table 2).

**Table 2:** Risk factors for functional dependence at 12, 24 and 36 months after first-ever IS stroke, adjusted relative risks, 95%confidence intervals and p-values (n = 303).

		12 months		24 months		36 months	
		Adj RR (95% CI)	p-value	Adj RR (95% CI)	p-value	Adj RR (95% CI)	p-value
Age (years)	≥ 65 *vs* < 65	1.88 (0.83–4.23)	0.128	3.01 (0.95–9.53)	0.061	3.08 (0.82–11.57)	0.095
Sex	female vs male	1.28 (0.53–3.09)	0.579	1.23 (0.39–3.93)	0.722	0.66 (0.17–2.52)	0.547
Years of education	≥ 8 *vs* < 8	0.81 (0.13–5.07)	0.821	0.22 (0.01–3.44)	0.280	0.59 (0.03–10.43)	0.719
Social Class ^β^	A / B *vs* D / E	2.66 (0.68–10.42)	0.161	3.25 (0.56–18.72)	0.188	1.84 (0.28–12.22)	0.529
	C *vs* D / E	1.94 (0.64–5.90)	0.244	1.94 (0.46–8.14)	0.368	2.22 (0.39–12.73)	0.373
Race	caucasian *vs* others	1.44 (0.46–4.50)	0.532	2.11 (0.40–11.07)	0.377	3.11 (0.34–28.25)	0.313
Hypertension	yes *vs* no	1.35 (0.55–3.32)	0.509	1.42 (0.45–4.50)	0.552	0.80 (0.22–2.83)	0.723
Diabetes	yes *vs* no	1.67 (0.66–4.24)	0.284	1.39 (0.43–4.53)	0.585	1.48 (0.34–6.40)	0.601
Smoking	yes *vs* no	1.62 (0.60–4.33)	0.338	3.23 (0.99–10.58)	0.053	2.81 (0.80–9.95)	0.108
Dyslipidemia Control	yes *vs* no	0.72 (0.28–1.85)	0.495	0.71 (0.22–2.27)	0.565	0.89 (0.23–3.38)	0.858
TOAST	LAA *vs* SAO	11.63 (1.31–103.21)	0.028	3.47 (0.55–21.73)	0.184	2.04 (0.36–11.68)	0.425
	CE *vs* SAO	19.80 (2.24–174.85)	0.007	11.82 (1.77–78.96)	0.011	5.29 (0.82–33.92)	0.079
	UND *vs* SAO	8.34 (0.95–73.24)	0.056	2.55 (0.40–16.10)	0.320	0.30 (0.03–3.29)	0.322
	Other *vs* SAO	16.68 (1.31–212.30)	0.030	4.38 (0.22–88.52)	0.336	2.49 (0.19–32.11)	0.486
Bamford	PACS *vs* LACS	1.10 (0.35–3.51)	0.868	0.50 (0.13–1.96)	0.316	1.75 (0.40–7.64)	0.459
	POCS *vs* LACS	2.07 (0.50–8.55)	0.314	0.36 (0.04–3.38)	0.372	0.63 (0.07–6.19)	0.694
	TACS *vs* LACS	3.68 (0.83–16.35)	0.087	0.97 (0.13–7.19)	0.973	4.38 (0.41–47.36)	0.224
Physical activity	yes *vs* no	0.32 (0.10–1.01)	0.052	0.32 (0.07–1.37)	0.124	0.33 (0.07–1.63)	0.174
Cardiopathy	yes *vs* no	1.49 (0.61–3.66)	0.380	0.59 (0.15–2.37)	0.459	1.27 (0.26–6.16)	0.770
NIHSS	Severe *vs* not severe	1.13 (0.34–3.82)	0.840	3.30 (0.62–17.64)	0.163	0.69 (0.08–5.93)	0.732

Adj RR: Adjusted Relative Risk; IS: Ischaemic Stroke

β Social class according to Brazilian Criteria of Economic Classification based on year 2013 National Household Sample Survey

LAA: large-artery oclusion; SAO: small-artery occlusion; CE: Cardioembolic; UND: Undetermined; PACS: Partial Anterior Circulation Syndrome; LACS: Lacunar Syndrome; TACS: Total Anterior Circulation Syndrome; POCS: Posterior Circulation Syndrome.

## Discussion

In this population-based study, we followed 367 patients with a first-ever stroke over 3 years. Approximately, between 20 to 40% had neurological sequelae in the first month and 15 to 20% in the first year. As expected, HS patients had a higher proportion of FD. Among IS incident patients, 60% were dependent at hospital admission, reducing to 33% at discharge and to 12% in the first year. Thereafter, we noticed a plateau in dependence proportion. Among the IS sample, patients with cardio-embolic, large artery atherosclerosis (LAA) and other determined IS subtypes had a significant and very high-adjusted risk of FD when compared to small-artery occlusion IS. Only cardio-embolic IS sustained this higher risk over the second year.

The burden of FD was quite distinct among stroke types’ survivors. Three years after incident stroke, FD was present in 10% for 198 IS survivors, 26% for 19 HS survivors and 7% for 15 SAH survivors. Although the proportion of FD among SAH patients were quite similar to IS patients their outcomes were not. Indeed, the 30-day case-fatality was 15% for IS, 46% for HS and 59% for SAH. Three years later, case-fatality was 35% for IS, 51% for HS and 55% for SAH patients. So, what driven the FD over observation time? Although causality measure is beyond our scope, our findings shows that prognosis of dependency was worse for HS and SAH than IS patients, mainly among patients who come to home or nursing home. In this subgroup, over 3 years, a third of all dependent IS were still dependent to their daily minor activities, whereas more than a half among HS and complete dependency among SAH patients.

Our overall 8% of dependency are lower than National Health Survey conducted in Brazil in 2013 which estimated 29.5% of men and 21.5% of women were functionally dependent [10]. Therefore, we estimate that Joinville (516,288 inhabitants in 2010) might have had 3,964 people with FD after stroke, with 3,290 (83%) due to IS, 396 (10%) to HS and 277 (7%) to SAH. These data are useful for public health policies, especially because there is no public rehabilitation in primary health units in Brazil [23]. However, even more important than the absolute numbers of incidence, we confirmed that most functional recovery occurs within the first months, and after that little further functional recovery can be expected. Therefore, stroke rehabilitation should start within the first year after a stroke [8,9].

Our findings are similar to other population-based studies, which reported data among stroke major types. A cohort with 3,773 first-ever stroke patients who were followed from 1995 to 2006 in the South London Stroke Register reported 110 per 1,000 stroke survivors were FD from 3 months to 10 years [6]. Of 418 stroke survivors in Auckland Stroke Outcome Study (2002–2003), 20 to 30% had moderate disability (requiring some help, but able to walk without assistance; mRS 3 to 5) 5–10 years following stroke [5]. A Swedish population-based study reported 28% of FD in the first year [24]. Higher rates were reported in Eastern Europe. In Belarus, for example, of the 269 survivors at 5 years, 52% (139) were disabled (modified Rankin score, ≥3) [25]. A large transversal study in France also showed higher proportions of FD after stroke. In a survey with 33,896 participants conducted in 2007 in France, 34% had and mRS of between 3 to 5 points [7].

We found an association between FD with aetiologic subtypes of stroke as classified by TOAST at 12 and 24 months, and anti-thrombotic therapy at 12 months. After 36 months, cardio-embolic subtype was no longer significantly associated to FD. It seems that the initial severity of stroke is not so relevant as time passes by, since other factors might interfere in the outcome, such as death in the case of more severe stroke patients.

This study has some limitations. Our registry does not perform ‘hot pursuit’ [26], which might result in an underestimation of stroke incidence. Although reliable and validated, functional assessments were performed by telephone, these are less ideal than face-to-face interviews [19]. We understand information bias in our sample is unlikely because our register had a trained nurse who used a validated scale for Rankin assessment by telephone call [20]. The strength of our data is the population-based sampling, which used ideal criteria of overlapping sources for stroke ascertainment [13].

Worldwide, a decrease in stroke mortality has been reported, which means an increase in the prevalence of disabled stroke survivors [1,6,7,10]. Despite the fact that stroke incidence in Joinville has decreased consistently over the last two decades, the proportion of dependent patients has not changed significantly [27]. This study shows that a city of a similar size to Joinville could expect 120 new patients every year who require help to perform daily practice activities. For public health policies, we can extrapolate, based on a recent prevalence national survey, that around 4,000 patients might have neurological disabilities and should receive public multidisciplinary rehabilitation. Moreover, these efforts should be focused in the first year. Cardio-embolic IS is a subgroup that deserves special attention. Long-term cohort studies are necessary as treatment improves and the maintenance of patients’ deficits over time will demand efficient and sufficient rehabilitation programmes [28].

## Supporting Information

S1 Table Joinville stroke database. (XLS)

S2 Table Trend-p value comparing the proportion of FD across time. (XLS)
